# Association of CYP2D6 and CYP2C19 metabolizer status with switching and discontinuing antidepressant drugs: an exploratory study

**DOI:** 10.1186/s12888-024-05764-6

**Published:** 2024-05-27

**Authors:** Jurriaan M. J. L. Brouwer, Klaas J. Wardenaar, Ilja M. Nolte, Edith J. Liemburg, Pierre M. Bet, Harold Snieder, Hans Mulder, Danielle C. Cath, Brenda W. J. H. Penninx

**Affiliations:** 1 Research School of Behavioral and Cognitive Neurosciences, University of Groningen, University Medical Center Groningen, Groningen, The Netherlands; 2 GGZ Drenthe Mental Health Center Drenthe, Assen, The Netherlands; 3 Department of Clinical Pharmacy, Wilhelmina Hospital Assen, Assen, The Netherlands; 4 Department of Clinical Pharmacy, Martini Hospital Groningen, Van Swietenlaan 1, Groningen, 9728 NT The Netherlands; 5 Department of Psychiatry, University Medical Center Groningen, Interdisciplinary Center Psychopathology and Emotion Regulation, University of Groningen, Groningen, The Netherlands; 6 Faculty of Behavioural and Social Sciences, University of Groningen, Groningen, The Netherlands; 7 Department of Epidemiology, University of Groningen, University Medical Center Groningen, Groningen, The Netherlands; 8 Rob Giel Research Center, University of Groningen, University Medical Center Groningen, Groningen, The Netherlands; 9 Department of Clinical Pharmacology and Pharmacy, Amsterdam University Medical Center, Vrije Universiteit Amsterdam, Amsterdam, The Netherlands; 10 Department of Psychiatry, Amsterdam University Medical Center, Vrije Universiteit Amsterdam, Amsterdam Public Health, Amsterdam, The Netherlands

**Keywords:** NESDA, *CYP2C19*, *CYP2D6*, Antidepressants, Switching, Discontinuing

## Abstract

**Background:**

Tailoring antidepressant drugs (AD) to patients’ genetic drug-metabolism profile is promising. However, literature regarding associations of ADs’ treatment effect and/or side effects with drug metabolizing genes *CYP2D6* and *CYP2C19* has yielded inconsistent results. Therefore, our aim was to longitudinally investigate associations between *CYP2D6* (poor, intermediate, and normal) and *CYP2C19* (poor, intermediate, normal, and ultrarapid) metabolizer-status, and switching/discontinuing of ADs. Next, we investigated whether the number of perceived side effects differed between metabolizer statuses.

**Methods:**

Data came from the multi-site naturalistic longitudinal cohort Netherlands Study of Depression and Anxiety (NESDA). We selected depression- and/or anxiety patients, who used AD at some point in the course of the 9 years follow-up period (*n* = 928). Medication use was followed to assess patterns of AD switching/discontinuation over time. *CYP2D6* and *CYP2C19* alleles were derived using genome-wide data of the NESDA samples and haplotype data from the PharmGKB database. Logistic regression analyses were conducted to investigate the association of metabolizer status with switching/discontinuing ADs. Mann–Whitney U-tests were conducted to compare the number of patient-perceived side effects between metabolizer statuses.

**Results:**

No significant associations were observed of CYP metabolizer status with switching/discontinuing ADs, nor with the number of perceived side effects.

**Conclusions:**

We found no evidence for associations between CYP metabolizer statuses and switching/discontinuing AD, nor with side effects of ADs, suggesting that metabolizer status only plays a limited role in switching/discontinuing ADs. Additional studies with larger numbers of PM and UM patients are needed to further determine the potential added value of pharmacogenetics to guide pharmacotherapy.

**Supplementary Information:**

The online version contains supplementary material available at 10.1186/s12888-024-05764-6.

## Introduction

Depressive and anxiety disorders are a major cause of disability and affect roughly 280 million and 301 million individuals worldwide respectively [1]. Antidepressant drugs (ADs) are widely prescribed for the treatment of depressive and anxiety disorders. However, the selection of an appropriate AD is mostly based on a process of trial and error, depending on perceived efficacy and tolerability of the AD. Treatment guidelines state that ADs should be switched, augmented with additional medication or discontinued when ineffective and/or not tolerated, mostly followed by a new treatment step [2]. Rush et al. (2006) showed that lower acute remission rates and higher relapse rates are to be expected when more treatment steps are required [3], indicating that it is important to identify an effective and tolerable pharmacotherapy in as few steps as possible. Gaining more insight into patient characteristics associated with primary effectiveness and tolerability of AD treatment could help to optimally tailor AD treatment to the patient’s needs [4]. However, reliable tailoring of ADs to the patient is a challenge, because of the large number of parameters involved in patients’ individual responses to ADs, such as age, gender, education and work status, amount of social support, smoking, and somatic and psychiatric comorbidities [5–7].

Another way to further tailor ADs to patients’ individual characteristics, is by matching prescribed medication to patients’ genetic profiles for drug metabolism [4]. Interindividual variability in efficacy and tolerability of antidepressants can be explained in part by genetic variability in the drug-metabolizing enzymes like cytochrome P450 isoforms in the liver [8, 9]. Two isoforms that are highly involved in the metabolism of ADs are Cytochrome P450 family 2 subfamily C member 19 (CYP2C19) [10] and Cytochrome P450 family 2 subfamily D member 6 (CYP2D6) [11]. The metabolizer status that reflects the rate at which an individual metabolizes a certain substrate can range between no metabolism (poor metabolizer; PM), reduced metabolism (intermediate metabolizer; IM), fully functional metabolism (normal metabolizer; NM), and increased metabolism (ultrarapid metabolizer; UM). This applies to both CYP2C19 and CYP2D6. Although the association of metabolizer status with therapeutic effects and side effects of ADs has been found to be inconsistent, and not to represent a one to one relationship, overall metabolizer status significantly affects plasma concentrations of ADs [12–18]. Whereas decreased metabolism is expected to increase plasma concentrations of ADs, increased metabolism is expected to decrease plasma concentrations of ADs [19]. Consequently, increased plasma concentrations may result in excess side effects due to relative ‘overdosing’, while decreased plasma concentrations may result in decreased or no effect of AD treatment [19].

Alternatively, although speculative, the association of metabolizer status with treatment persistence, discontinuing ADs and/or switching AD type can be investigated, where discontinuing and switching AD treatment could be interpreted as an expression of a lack of perceived treatment effect and/or low treatment tolerability. There is some evidence suggesting that CYP-metabolizer status is associated with treatment persistence, or switching or discontinuing AD treatment. One study showed that the risk of switching from escitalopram to another AD within one year was at least three times higher in *CYP2C19* PM and UM patients than in *CYP2C19 *NM patients [20]. Two studies found that the risk of switching ADs was up to five times higher in *CYP2D6* PM patients than in *CYP2D6 *NM patients [21, 22]. Finally, one prospective intervention study showed that switching between ADs was more prevalent in patients taking ADs that were not in line with recommendations based on their *CYP2C19* and *CYP2D6* metabolizer status [23] for gene-drug interactions according to the Clinical Pharmacogenetics Implementation Consortium (CPIC) and the Dutch Pharmacogenetics Working Group (DPWG) [24–27].

To conclude, the number of longitudinal studies on this topic is relatively limited and focused on either CYP2D6 or CYP2C19. Therefore, we first aimed to investigate if and how both *CYP2D6* and *CYP2C19* metabolizer status are longitudinally associated with switching or discontinuing ADs that are substrates for these CYP enzymes. Further, we aimed to explore whether the number of perceived side effects differed between patients depending on their metabolizer status. We used data from a large cohort of patients with depressive and anxiety disorders who were followed for nine years. Therefore, our first hypothesis was that *CYP2D6/CYP2C19* PM and IM patients, in addition to *CYP2C19* UM patients, would show higher rates of switching and/or discontinuing AD treatment over time than *CYP2D6/CYP2C19* NM patients. Our second hypothesis was that *CYP2D6/CYP2C19* PM and IM patients would perceive relatively more side effects compared with *CYP2D6/CYP2C19* NM patients.

## Materials and methods

### Study sample

Data were obtained from the Netherlands Study of Depression and Anxiety (NESDA), a multi-site naturalistic longitudinal cohort study among 2,981 adults (18–65 years old), in which the long-term course and consequences of depressive and anxiety disorders are investigated. The cohort consists of adults with a *current* diagnosis of a depressive and/or anxiety disorder (*n* = 1,701) according to DSMIV criteria, adults with a *life-time* diagnosis, or at increased risk due to a family history of anxiety and/or depression, or with *subthreshold* depressive or anxiety symptoms (*n* = 907), and healthy controls (*n*= 373). The rationale, objectives, and methods are extensively described elsewhere [28]. Patients with various depressive (major depressive disorder or dysthymia) and anxiety disorders (generalised anxiety disorder, panic disorder, agoraphobia, and social anxiety disorder) were recruited from the community, primary care, and specialized mental healthcare settings. They were invited for assessments on a regular basis, each of which included face-to-face interviews, self-report questionnaires, and medical examinations. Baseline assessments took place between 2003 and 2007, with follow-up (FU) assessments taking place at 1, 2, 4, 6, and 9 years after baseline.

All participants signed informed consent. NESDA’s study protocol was approved centrally by the Ethical Review Board of VU University Medical Center and locally by the review board of each participating center (METC number 2003–183). All study procedures were carried out in accordance with the Declaration of Helsinki.

### Measures

#### Genotyping procedure

Genome-wide genotyping was performed using either the Perlegen-Affymetrix 5.0, or Affymetrix 6.0 genotyping chip and genotypes were called with BirdSeed (Affymetrix, Inc., Santa Clara, CA, USA). Single Nucleotide Polymorphisms (SNPs) were removed if they could not be adequately mapped, call rate < 95%, MAF < 0.01, HWE *p*-value < 10^–5^, allele frequency difference with 1000Genomes reference > 20%, or palindromic SNPs with allele frequency > 35%. Samples were excluded in the case of call rate < 90%, deviant heterozygosity (abs(PLINK F) > 0.1), sex mismatch, unexpected first-degree relatedness, or being non-European. Whole-genome imputation was performed with Impute software using the 1000Genomes Phase 1 Integrated Release 3 ALL reference panel [29]. After imputation, SNPs within a 10kb distance from the *CYP2D6* and *CYP2C19* genes with a maximum posterior probability > 90% were selected for further imputation of *CYP* alleles. To create a reference panel, two databases were downloaded, one for *CYP2D6* and one for *CYP2C19,* constructed by PharmGKB [30, 31], which recorded what combinations of genetic variants (i.e. haplotypes) constitute which *CYP* allele. From these databases, we selected SNPs present in NESDA and those that best predicted our *CYP* alleles of interest, see Supplementary Table 1, Additional file 1. Unfortunately, the available data did not allow us to identify the gene deletion (CYP2D6*5), which can translate to *CYP2D6* IM or PM status, nor duplications of *CYP2D6* alleles, which can translate to *CYP2D6* UM status. As a result, we were unable to identify patients with a *CYP2D6* IM or PM metabolizer status due to a CYP2D6*5 gene deletion and patients with a *CYP2D6* UM metabolizer due to duplications of *CYP2D6* alleles. Instead, patients who had duplications of *CYP2D6* alleles or gene deletion (CYP2D6*5) were assigned a *1 allele. We expect this to be 1–2% and 5% of our study sample respectively, based on UM prevalence and *5 allele frequency in the Netherlands, see Supplementary Table 2, Additional file 2. Using the constructed reference panels, *CYP* alleles for *CYP2D6* and *CYP2C19 *were imputed using IMPUTE v2 [32]. Only alleles that were imputed with a maximum posterior probability > 0.9 were converted to obtain the most likely genotype and included in further analyses.

#### Genotype translation to metabolizer status

Supplementary Table 1, Additional file 1 shows the alleles of interest that could be identified for *CYP2D6* and *CYP2C19* during the genotyping procedure, in which each allele has its own metabolic capacity. For *CYP2D6*, *1 and *2 are fully functional, while *3, *4, and *6 are inactive, and *10, *17, and *41 have a reduced functionality [33]. The metabolizer status (PM, IM, and NM) was defined based on the total gene dose of the genotype (Supplementary Table 3, Additional file 3). Here, the gene dose of a fully functional, reduced functional and inactive allele is 1, 0.5 and 0 respectively. PM was defined as having a gene dose of 0, IM was defined as having a gene dose of 0.5–1, NM was defined as having a gene dose of 1.5–2.5 [34]. For *CYP2C19*, *1 is fully functional, *2 and *3 are inactive, and *17 has an increased functionality [35]. The metabolizer status (PM, IM, NM, and UM) was defined according to the definition of the DPWG (Supplementary Table 3, Additional file 3). Here, patients with two inactive alleles or two alleles with reduced functionality were defined as PM. Patients with one inactive allele or allele with reduced functionality were defined as IM. Patients with two fully functional alleles or one fully functional allele and one allele with increased functionality were defined as NM. Patients with two alleles with increased functionality were defined as UM.

#### Medication use

**Fig. 1:**
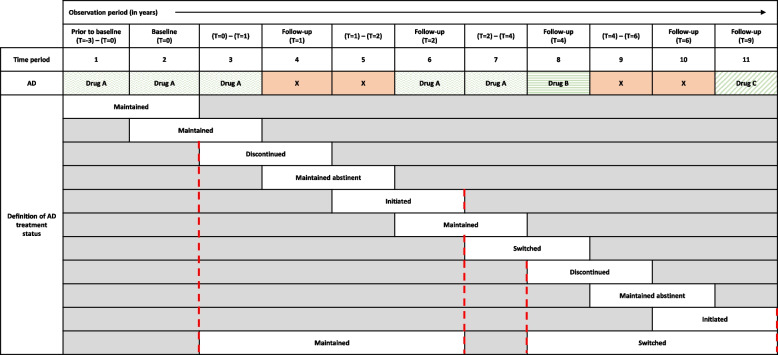
A schematic illustration of maintained, discontinued, initiated, switched, or maintained abstinence of AD treatment during the observation period. Definitions of AD treatment status based on two time periods interspersed with one or more time periods with no AD treatment (see red dotted lines) are preferred over definitions of AD treatment status based on two consecutive time periods. AD: antidepressant drug

Study outcomes (switching or discontinuing an AD) were based on data collected for the period starting three years prior to baseline up to nine years after baseline (the *observation period*). During this period, patients had six assessments (at baseline and at 1-, 2-, 4-, 6-, and 9-year FU). During all assessments, except for the 9-year FU, we assessed both current and previous medication use. This resulted in a total of 11 subsequent medication use assessments across the observation period. At baseline, medication up to three years prior to baseline (time period 1) and current medication (time period 2) were assessed. Medication use between baseline and 1-year FU (period 3), current use at 1-year FU (period 4), between 1-year and 2-year FU (period 5), current use at 2-year FU (period 6), between 2-year and 4-year FU (period 7), current use at 4-year FU (period 8), between 4-year and 6-year FU (period 9), current use at 6-year FU (period 10), and current medication use at 9-year FU (period 11) were assessed (see Fig. 1 for an overview).

For each assessment, patients were asked to bring containers of all current medication. If the containers were absent, information on the active component or medication brand, the dose, and frequency of intake was obtained by interview. The ADs used were classified using the World Health Organization Anatomical Therapeutic Chemical (ATC) classification system (Table 1). The following categories were identified: non-selective monoamine reuptake inhibitors, selective serotonin reuptake inhibitors, non-selective monoamine oxidase inhibitors, monoamine oxidase A inhibitors, and a miscellaneous group of antidepressant drugs. Each identified AD could be metabolized by CYP2D6, CYP2C19, or by both (Table 1) [34, 36].

**Table 1:** Overview of antidepressant drugs that are substrates for CYP2D6 and/or CYP2C19

CYP2C19	CYP2D6	CYP2C19 and CYP2D6
Agomelatine^a^ (N06AX22)	Desipramine (N06AA01)	Amitriptyline (N06AA09)
Clomipramine (N06AA04)	Duloxetine (N06AX21)	Citalopram (N06AB04)
Escitalopram (N06AB10)	Fluoxetine (N06AB03)	Doxepin (N06AA12)
Moclobemide (N06AG02)	Fluvoxamine (N06AB08)	Imipramine (N06AA02)
Sertraline^a^ (N06AB06)	Maprotiline^a^ (N06AA21)	
	Mianserin^a^ (N06AX03)	
	Mirtazapine^a^ (N06AX11)	
	Nortriptyline (N06AA10)	
	Paroxetine (N06AB05)	
	Trazodone^a^ (N06AX05)	
	Venlafaxine (N06AX16)	
	Vortioxetine^a^ (N06AX26)	

^a^ Based on “Farmacogenetica *–* https://www.knmp.nl” [34]

#### Definition of AD treatment status over time

To define a patient as discontinued, switched, or maintained on AD treatment, we compared either two consecutive time periods or periods that were interspersed with one or more time periods with no AD treatment. Figure 1 illustrates this process. At first, AD treatment per two consecutive time periods was defined throughout the observation period. However, two non-consecutive time periods with AD treatment, interspersed by one or more time periods with no AD treatment, were compared as well. When compared, the definitions of AD treatment status based on two consecutive time periods within this time interval were neglected (see red dotted lines in Fig. 1).

#### Definition of patients who discontinued, switched, or maintained AD treatment

A patient was defined as *maintained on AD* when an AD was used somewhere during time periods 1–10 and no switch took place during the observation period. A patient was defined as *discontinued *when an AD was used somewhere during time periods 1–4, but not during time periods 5–11, and no switch took place during the observation period. The cut-off point between time periods 1–4 and 5–11 was based on a recommended AD-treatment duration of 6–12 months after a first or recurrent depressive episode [37]. A patient was defined as *switched* if AD treatment was switched somewhere during the observation period. See Supplementary Fig. 1, Additional file 4 for a schematic presentation of these three definitions. A dichotomous outcome variable was constructed that contrasted *discontinued* patients (coded as 1) with *maintained* patients (coded as 0). A second variable was created to contrast *switched* patients (coded as 1) with *maintained* patients (coded as 0).

#### Reasons for discontinuing treatment with antidepressant drugs

During the assessments at time periods 5 (1-year to 2-year FU), 7 (2-year to 4-year FU), and 9 (4-year to 6-year FU), patients were asked, where applicable, why they had discontinued a certain AD. The reasons patients could select from, were: (a) “being symptom-free”, (b) “treatment was ineffective”, or (c) “treatment resulted in too many side effects”. However, there are patients that – after discontinuing AD treatment – may have restarted drug therapy with a different AD (switching) during the following time period.

To investigate the association between metabolizer statuses and reasons for discontinuing AD treatment, the following steps were performed. First, patients were selected who switched or discontinued AD(s) at time periods 6 (2-year FU), 8 (4-year FU), and 10 (6-year FU). This was based on a direct comparison in AD treatment with the previous time periods 5 (1-year to 2-year FU), 7 (2-year to 4-year FU), and 9 (4-year to 6-year FU) respectively*.* In the next step, a variable was created that categorized patients into single subgroups according to whether the AD they used was a substrate for CYP2D6, CYP2C19, or both (Table 1). To enable this specific subgrouping, patients were selected as illustrated in Supplementary Fig. 2, Additional file 5. Next, for each AD subgroup, dichotomous outcome variables were created for the reasons for discontinuing AD treatment. One variable contrasted “treatment was ineffective” (coded as 1) with “being symptom-free” (coded as 0) and a second variable contrasted “treatment resulted in too many side effects” (coded as 1) with “being symptom-free” (coded as 0).

#### Number of perceived side effects

The number of perceived side effects related to AD use were assessed between baseline and 6 years FU (at time period 3, 5, 7, and 9) with the 12-question self-report Antidepressant Side Effect Checklist (ASEC-12 [38]). The ASEC-12 items cover insomnia, daytime sleepiness, restlessness, muscle spasms/twitching, dry mouth, profuse sweating, sexual disorders, nausea, constipation, diarrhoea, weight gain, and dizziness [38], which were rated on a 2-point response scale (0 = “reported”; 1 = “not reported”). Side effects were divided into three categories: serotonergic (insomnia, restlessness, muscle spasms/twitching, profuse sweating, sexual disorders, nausea, and diarrhoea), cholinergic (dry mouth and constipation), and histaminergic (daytime sleepiness and weight gain) [39].

To investigate the difference in perceived total, serotonergic, cholinergic, and histaminergic side effects between metabolizer statuses for both CYP2C19 and CYP2D6, we first created a variable per AD that indicated the number of perceived side effects for that particular AD. Second, variables were created for *CYP2C19* NM, IM, and PM patients, and for *CYP2D6* NM, IM, and PM patients, indicating the number of perceived side effects of the AD as a substrate for CYP2C19, CYP2D6, or both. When a patient used more than one AD that was a substrate for CYP2C19, CYP2D6, or both, the average number of side effects was calculated. Lastly, we created a variable for each metabolizer status of CYP2C19 and CYP2D6, reflecting the average perceived side effects per patient over the four time periods.

### Sample selection

**Fig. 2:**
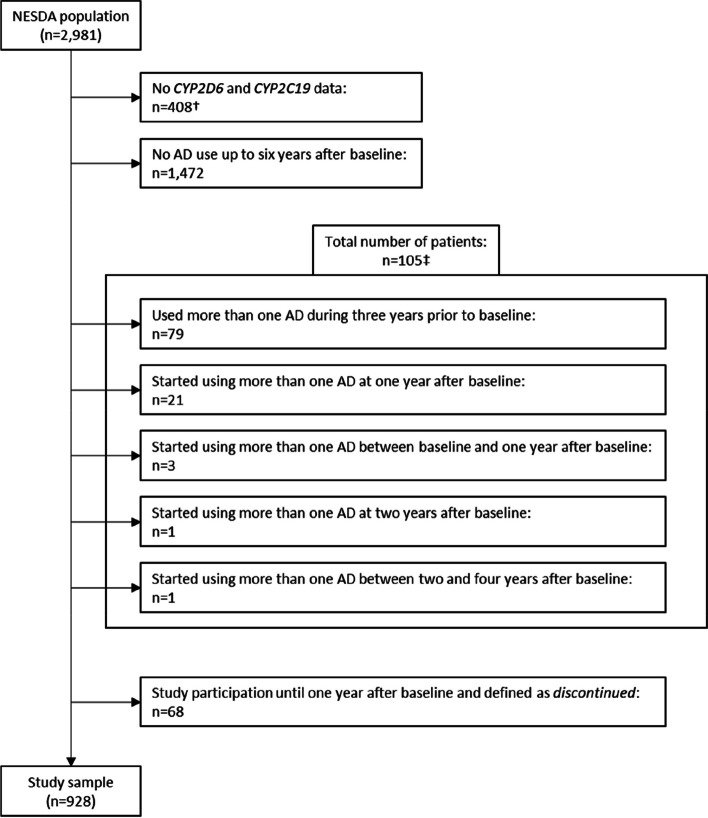
Flowchart indicating the selection process. †: patients with no GWAS data or for whom imputation was inadequate for both *CYP2C19* and *CYP2D6*; ‡: patients who either used more than one AD three years prior to baseline (time period 1) or who started using more than one AD during the observation period (time period 2–10) with no AD use in the previous time periods

The selection process is illustrated in Fig. 2. For this study, we only selected patients with *CYP2D6* and *CYP2C19* genetic data who used a single AD that was either a substrate of CYP2D6, CYP2C19, or both. Thus, only patients were selected who either used a single AD three years prior to baseline (time period 1) or who started a single AD during the observation period (time period 2–10) with no AD use in the previous time periods. Patients were systematically selected per time period (1 through 10), as illustrated in Supplementary Fig. 2, Additional file 5. Throughout the observation period, some patients were lost to follow-up. Seventy-nine patients participated in the study during time periods 1–4. Of these, 68 patients were defined as *discontinued*, as they did not use AD during time periods 5–11 (criterion 2). However, as no information about continued use after period 4 is available, these patients could not be reliably classified and were therefore not selected. This resulted in a final study sample of 928 patients.

### Statistical analyses

All statistical analyses were performed with IBM SPSS Statistics version 26.0 for Windows (Armonk, New York, USA). First, descriptive analyses were conducted. Means and standard deviations were calculated for normally distributed continuous variables. Medians and interquartile ranges (IQR) were calculated for continuous variables that were not normally distributed. Frequencies and percentages were calculated for categorical variables.

#### Association of metabolizer status with switching or discontinuing AD treatment

Logistic regression analyses were conducted to investigate the association of CYP metabolizer status (independent variable) with the dependent variables of switching (0/1) and discontinuing (0/1). This analysis was conducted separately in subsamples of either CYP2C19 or CYP2D6 substrate-related AD users. Patients treated with an AD that was a substrate of both CYP2C19 and CYP2D6 were included in both subsamples.

#### Comparison of perceived side effects between metabolizer statuses

A Mann–Whitney U test was used to test the difference in distributions of perceived total, serotonergic, cholinergic, and histaminergic side effects (averaged across assessments) between metabolizer statuses for both CYP2C19 and CYP2D6.

## Results

### Study sample

Table 2 presents the characteristics of the study sample (*n* = 928). Patients were classified as *CYP2C19* PM (2.5%; *n* = 23), IM (20.9%; *n* = 194), NM (70.7%; *n* = 656), and UM (5.9%; *n* = 55). In addition, they were classified as *CYP2D6* PM (2.4%; *n* = 22), IM (14.2%; *n* = 132), and NM (81.3%; *n* = 754). In 2.2% (*n* = 20) of the study sample, imputation quality was insufficient to establish a *CYP2D6* metabolizer status. Considering that the complete study sample is of North-European ancestry, the prevalences of *CYP2C19* PM, IM, NM, and UM in the current study are similar to those presented in the Netherlands (Supplementary Table 4, Additional file 6). However, for *CYP2D6*, whereas the prevalences of PM and IM patients are relatively lower, compared with the prevalences presented in the Netherlands, it is higher for NM patients (Supplementary Table 2, Additional file 2). Table 3 presents the distribution of AD subgroups in the total study sample, and in the subsamples of CYP2D6 and CYP2C19 substrate users. Of the study sample (*n* = 928), 68.6% (*n* = 636) used selective serotonin reuptake inhibitors (SSRIs), while 10.2% (*n* = 95) used non-selective monoamine reuptake inhibitors, such as tricyclic antidepressants (TCAs). In addition, 20.9% (*n* = 194) used an AD of the miscellaneous group of ADs, such as serotonin and norepinephrine reuptake inhibitors (SNRIs) (*n* = 123). Finally, 0.2% (*n* = 2) used monoamine oxidase A inhibitors and 0.1% (*n* = 1) used a non-selective monoamine oxidase inhibitor.

**Table 2:** Baseline characteristics of the study group

	**Study sample (** ***n*** ** = 928)**
**North-European ancestry**, n (%)	928 (100%)
**Age (years)**, mean | SD	42 | 12
**Gender**, n (%)	
Male	304 (32.8%)
Female	624 (67.2%)
**Level of education**, n (%)	
Low	68 (7.3%)
Intermediate	565 (60.9%)
High	295 (31.8%)
**Lifetime disorder status**, n (%)	
Healthy control	22 (2.4%)
Lifetime depressive or anxiety disorder	906 (97.6%)
**Presence of lifetime depressive disorders**, n (%)	
No dysthymia, no MDD	108 (11.6%)
Dysthymia, no MDD	13 (1.4%)
MDD, no dysthymia	521 (56.1%)
Dysthymia and MDD	286 (30.8%)
**Number of MDD episodes**, median (IQR)	1 (1 – 4)
**Presence of lifetime anxiety disorders**, n (%)	
No anxiety diagnosis	186 (20.0%)
Social phobia	408 (44.0%)
Panic with agoraphobia	269 (29.0%)
Panic without agoraphobia	147 (15.8%)
Agoraphobia	121 (13.0%)
Generalised Anxiety Disorder (GAD)	335 (36.1%)
**Number of anxiety diagnoses (past 6 months)**, median (IQR)	1 (0 – 2)

*IQR* Interquartile range, *MDD* Major depressive disorder,* SD* Standard deviation

**Table 3:** Number and percentage of patients per AD and CYP status group

**Antidepressant subgroup**	**Study sample (** ***n*** ** = 928)** n (%)	**CYP2D6 substrate users (** ***n*** ** = 796)** n (%)	**CYP2C19 substrate users (** ***n*** ** = 313)** n (%)
Amitriptyline	53 (5.7%)	52 (6.5%)	53 (16.9%)
Citalopram	146 (15.7%)	144 (18.1%)	146 (46.6%)
Clomipramine	33 (3.6%)	–	33 (10.5%)
Duloxetine	9 (1.0%)	9 (1.1%)	–
Escitalopram	18 (1.9%)	–	18 (5.8%)
Fluoxetine	77 (8.3%)	76 (9.5%)	–
Fluvoxamine	53 (5.7%)	51 (6.4%)	–
Imipramine	2 (0.2%)	2 (0.3%)	2 (0.6%)
Maprotiline	2 (0.2%)	2 (0.3%)	–
Mianserin	2 (0.2%)	2 (0.3%)	–
Mirtazapine	63 (6.8%)	63 (7.9%)	–
Moclobemide	2 (0.2%)	–	2 (0.6%)
Nortriptyline	5 (0.5%)	5 (0.6%)	–
Paroxetine	283 (30.5%)	275 (34.5%)	–
Sertraline	59 (6.4%)	–	59 (18.8%)
Tranylcypromine	1 (0.1%)	–	–
Trazodone	4 (0.4%)	4 (0.5%)	–
Tryptophan	2 (0.2%)	–	–
Venlafaxine	114 (12.3%)	111 (13.9%)	–
**Patient definition** ^a^			
Maintained	479 (51.6%)	412 (51.8%)	174 (55.6%)
Switched	328 (35.3%)	274 (34.4%)	104 (33.2%)
Discontinued	121 (13.0%)	110 (13.8%)	35 (11.2%)

^a^ Observed over a 9-year period

### Association of metabolizer status with switching or discontinuing AD treatment

Table 3 presents the number and percentage of patients who maintained, switched or discontinued AD treatment during the observation period: 51.6–55.6% of patients maintained AD treatment during the observation period, while 33.2–35.3% switched and 11.2–13.8% discontinued AD treatment during the observation period. Logistic regression analyses (Table 4) indicated that there were no significant associations of *CYP2C19* and *CYP2D6* metabolizer status with switching versus maintained AD treatment and with discontinued versus maintained AD treatment.

**Table 4:** Association of *CYP2D6* and *CYP2C19* metabolizer status with switching/discontinuing AD treatment over time

	**Maintained AD use**	**Discontinued AD use**		**Switched AD use**	
***CYP2D6***	**n (%)**	**n (%)**	**Discontinued vs. maintained AD OR (95% CI) (** ***n*** ** = 168)** ^a^	**n (%)**	**Switched vs. maintained AD** **OR (95% CI) (** ***n*** **= 274)** ^a^
*NM (n* = *663)*	352 (53.1%)	88 (13.3%)	1 (reference)	223 (33.6%)	1 (reference)
*IM/PM (n* = *133)*	60 (45.2%)	22 (16.5%)	1.47 (0.85 – 2.52)	51 (38.3%)	1.34 (0.89 – 2.02)
***CYP2C19***	**n (%)**	**n (%)**	**Discontinued vs. maintained AD OR (95% CI) (** ***n*** ** = 51)** ^b^	**n (%)**	**Switched vs. maintained AD** **OR (95% CI) (** ***n*** **= 104)** ^b^
*NM (n* = *211)*	120 (56.8%)	24 (11.4%)	1 (reference)	67 (31.8%)	1 (reference)
*UM (n* = *26)*	13 (50.0%)	4 (15.4%)	1.54 (0.46 – 5.13)	9 (34.6%)	1.24 (0.50 – 3.05)
*IM/PM (n* = *76)*	41 (54.0%)	7 (9.2%)	0.85 (0.34 – 2.13)	28 (36.8%)	1.22 (0.70 – 2.15)

^a^ Reference: maintained users (*n = 412*), ^b^ Reference: Maintained users(*n = 174), AD *Antidepressant drug*, CI *Confidence interval*, IM *Intermediate metabolizer*, NM *Normal metabolizer*, OR *Odds ratio*, PM *Poor metabolizer*, UM *Ultrarapid metabolizer

### Reasons for discontinuing AD treatment

Overall, we had information on the reasons for discontinuing AD treatment in *n* = 182 patients. Table 5 presents the number of patients who, at time periods 5, 7, or 9, switched or discontinued AD treatment and discontinued AD treatment because they were symptom-free, because of ineffective treatment or because treatment resulted in too many side effects across CYP enzymes and metabolizer status. Overall, most patients discontinued AD treatment because they felt they were symptom-free. Given the small number of patients, no additional comparative analysis was conducted with this study sample.

**Table 5:** Reasons for discontinuing AD treatment over time, stratified by *CYP2D6* and *CYP2C19* metabolizer status

	**Symptom-free**	**Treatment was ineffective**	**Treatment-induced side effects**
***CYP2D6***	**n (%)**	**n (%)**	**n (%)**
*NM (n* = *129)*	48 (37.2%)	33 (25.6%)	48 (37.2%)
*IM/PM (n* = *29)*	12 (41.4%)	9 (31.0%)	8 (27.6%)
***CYP2C19***	**n (%)**	**n (%)**	**n (%)**
*NM (n* = *49)*	20 (40.8%)	12 (24.5%)	17 (34.7%)
*IM/PM/UM (n* = *23)*	8 (34.8%)	5 (21.7%)	10 (43.5%)

*IM *Intermediate metabolizer*, NM *Normal metabolizer*, PM *Poor metabolizer*, UM *Ultrarapid metabolizer

### Comparison of perceived total side effects between metabolizer statuses

Mann–Whitney U tests indicated no significant differences in the distributions of perceived total, serotonergic, cholinergic, and histaminergic side effects between NM patients and IM/PM patients, for both CYP2C19 and CYP2D6 substrates.

### Power calculations

Considering the known low prevalence of UM and PM patients for both CYP2C19 and CYP2D6 in Europe, we performed a post hoc power calculation with G*Power 3.1 software, of which the results can be found in Supplementary Material, Additional file 7. Compared with a standard power of 0.8, the calculated power range for the associations between *CYP2C19* metabolizer status and switching/discontinuing AD treatment was 0.06–0.13, while this was 0.27–0.29 for *CYP2D6* metabolizer status.

## Discussion

This study investigated if and how *CYP2D6* and *CYP2C19* metabolizer status of patients with depressive and/or anxiety disorders was associated with 1) switching or discontinuing AD treatment (CYP2D6 substrate users: *n* = 796); CYP2C19 substrate users: *n* = 313), and 2) whether there was a significant difference in perceived total side effects between patients with a different metabolizer status. The results showed no associations between *CYP2D6* IM/PM metabolizer status and switching (OR [95% CI]: 1.34 [0.89–2.02]) or discontinuing (OR [95% CI]: 1.47 [0.85–2.52]) AD treatment. In addition, no associations were found between *CYP2C19* UM metabolizer status and switching (OR [95% CI]: 1.24 [0.50–3.05]) or discontinuing (OR [95% CI]: 1.54 [0.46–5.13]), nor between *CYP2C19* IM/PM metabolizer status and switching (OR [95% CI]: 1.22 [0.70–2.15]) or discontinuing (OR [95% CI]: 0.85 [0.34–2.13]). Furthermore, no significant differences were found in perceived total side effects between patients with *CYP2D6* and *CYP2C19* metabolizer status who used ADs. Finally, the reason for discontinuing AD treatment, was mostly because patients felt they were symptom-free, rather than them experiencing ineffective treatment or treatment-induced side effects.

Our finding that *CYP2C19* and *CYP2D6* metabolizer status was not significantly associated with switching or discontinuing ADs does not align with some previous studies. One small retrospective study (*n* = 77) by Mulder et al. (2005) showed that *CYP2D6* PM patients (*n* = 10) were more likely to switch ADs compared with *CYP2D6* NM patients (*n* = 67) during an observation period of approximately 4.5 years [22]. A possible explanation for the discrepancy with Mulder’s study might lie in the retrospective nature of Mulder et al.’s study (2005), or in the relatively low power of the current study as presented in Supplementary Material, Additional file 7. Another retrospective study by Jukić et al. (2018) showed that *CYP2C19* PM (*n* = 88; 4%) and UM patients (*n* = 604; 28%) were more likely to switch from escitalopram to another AD compared with NM patients (*n*= 837; 39%) [20]. Whereas the prevalence of *CYP2C19* UM patients in the current study was *n* = 55 (5.9%), which is in line with the prevalence indicated in Europe (3.2–7.3%), in the study by Jukić et al. (2018) this prevalence was 28.5% [20]. This is because in Jukić et al.’s study (2018), the definition of *CYP2C19* UM patients includes *CYP2C19* *1/*17 patients, which is based on the CPIC guidelines [27], whereas the definition of *CYP2C19* UM in the current study is based on the DPWG guidelines [27], which define *CYP2C19* *1/*17 patients as NM patients. Still, after redefining *CYP2C19* *1/*17 patients as *CYP2C19* UM patients (*n* = 302; 32.5%) and reanalysing the association of *CYP2C19* metabolizer status with switching/discontinuing AD treatment over time, no significant associations were found (data available from the first author upon request). The finding by Jukić et al. (2018) that UM patients were more likely to switch from escitalopram to another AD when compared with NM patients, may lie in the subtherapeutic plasma concentration seen in these UM patients. In the current study, only 5.8% (*n* = 18) of the CYP2C19 substrate users (*n* = 313) used escitalopram, whereas 46.6% (*n* = 146) used citalopram, an AD in which lower plasma concentrations may be less clinically relevant [24]. This may have impaired the possibility of finding a significant association between *CYP2C19* UM patients and increased odds of switching/discontinuing AD treatment. The finding that there were no differences between IM and PM patients in perceived total side effects compared with NM patients is supported by previous literature. Hodgson et al. (2015) found that *CYP2C19* and *CYP2D6* metabolizer status did not predict the total number of self-reported side effects of nortriptyline and escitalopram use over a 12-week period in 251 and 340 patients respectively [40]. Conversely, Zastrozhin et al. (2018) found that 17 patients with reduced CYP2D6 enzyme activity had lower tolerability at day 9 and 16 of fluvoxamine treatment, compared with 29 *CYP2D6* NM patients [41]. A meta-analysis by Fabbri et al. (2018a) presented a similar finding, namely that *CYP2C19* PM patients were at higher risk of side effects during the first four weeks of citalopram/escitalopram treatment, although this effect diminished after two months [42]. These results suggest that generally associations found between reduced CYP-enzyme activity and lower tolerability of ADs might be limited to the first weeks of treatment. This would be in line with our findings, considering our study design, in which we only had long-term (at least one year) follow-up data on medication use and tolerability. The available data did not allow us to pick up short-term tolerability changes related to potential differences between metabolizer status groups. Moreover, in the current study, it was unclear when exactly AD treatment was initiated or switched prior to the yearly assessments. Further, with the large assessment intervals, ranging between one and three years, PM and/or IM patients might have already been assigned to more tolerable dose regimens prior to the assessments by treating clinicians. This may have blurred potential differences in the number of perceived side effects between metabolizer statuses. However, upon comparing daily defined dosages (DDD) between metabolizer statuses, no significant differences were found (data available from the first author upon request). Another possible explanation for the lack of differences in observed findings between metabolizer statuses might be phenoconversion by co-medication. Inhibition of enzymatic capacity by co-medication, such as inhibition of CYP2D6 by paroxetine or fluoxetine, may cause increased plasma concentrations of the drug of interest, which may result in increased risk of side effects [43, 44]. Our data did not allow us to investigate this.

The current study has several strengths, including the long follow-up period and its systematic and highly standardized measurement procedures. And although sample size was relatively larger than previous studies, power was insufficient to draw definite conclusions. Furthermore, several study limitations should be kept in mind when interpreting the current findings. First, we were unable to identify duplications of *CYP2D6* alleles nor the gene deletion CYP2D6*5 and were therefore unable to identify patients with a *CYP2D6* UM or CYP2D6*5 IM or PM metabolizer status respectively. Alleles that could not be identified were assigned a *1 allele. Therefore, we cannot rule out that we might have overestimated the prevalence of *CYP2D6* NM and underestimated the prevalence of *CYP2D6* IM and/or PM patients in the current study, in addition to not being able to identify *CYP2D6* UM patients altogether. In fact, the prevalence of *CYP2D6* IM (14.2%) and PM (2.4%) patients in the study sample was relatively lower than those presented in the Netherlands (IM: 37.8–40%; PM: 5.5–6.6%; NM: 51.4–55.7%), while the prevalence of *CYP2D6* NM (81.3%) patients was relatively higher, see Supplementary Table 2, Additional File 2. Second, with NESDA being a naturalistic cohort with at least yearly assessments of depressive and/or anxiety symptoms, we were unable to pick up on short-term effects or side effects when starting, switching or discontinuing IM, PM, and/or UM patients either before or during the study. Furthermore, theoretically some patients may already have been prescribed an appropriate AD or dose regimen based on their metabolizer status prior to study entry (although pre-emptive screening is not established practice in the Netherlands), or during the observation period through, for example, therapeutic drug monitoring of TCAs. As we lacked dose regimen data for the greater part of the observation period, potential metabolizer status effects that were operant might have been blurred. Third, previous literature has indicated a significant plasma concentration-effect/side effect relationship of some ADs, including nortriptyline and imipramine. However, significant plasma concentration-effect/side effect relationships are not established for all ADs, such as selective serotonin reuptake inhibitors (SSRIs) [43, 45, 46]. This means that alterations in plasma concentrations, due to increased/reduced enzymatic capacity, may be less clinically relevant in SSRIs, when compared with TCAs. More specifically, with SSRIs, higher plasma concentrations might not always result in more treatment efficacy. This is supported by Meyer et al. (2004), who showed that a 80% serotonin transporter (SERT) occupancy was achieved at minimum therapeutic SSRI doses, which plateaued at higher doses [47, 48]. Furthermore, SSRIs have a broad therapeutic index, which is a quantitative measurement of the relative safety of a drug. This indicates that SSRIs are relatively tolerable ADs. In our study sample, only 10% (*n* = 95) used TCAs, while most patients (69%; *n* = 686) used an SSRI. This is 71% of *CYP2C19* IM/PM/UM patients and 62% of *CYP2D6 *IM/PM patients. Overall, an increase in SSRI plasma concentration in IM and/or PM patients may still be well tolerated and/or may not lead to relatively more treatment effect, possibly explaining the lack of significant findings in the current study. A fourth limitation is that the current results are based on retrospective assessments of perceived side effects and medication-use over longer periods of time, which may have caused some recall-bias. A fifth limitation, considering the known low prevalence of UM and PM patients for both CYP2C19 and CYP2D6 in Europe, is the risk of the total sample size being underpowered as a result of which some potentially relevant associations may have been missed. However, we did not perform an a priori power calculation, because data on expected proportions of switched/discontinued cases per metabolizer status was rather limited, for both CYP2D6 and CYP2C19 [20–22]. Instead, we performed a post hoc power calculation with G*Power 3.1 software [49], of which the results can be found in Supplementary Material, Additional file 7. Overall, we concluded that the current study was underpowered to adequately investigate associations between *CYP2C19* (calculated power range: 0.06–0.13) and *CYP2D6* (calculated power range: 0.27–0.29) metabolizer status and switching/discontinuing AD treatment. When redefining *CYP2C19* UM patients according to the CPIC guidelines, calculated power ranged from 0.05 to 0.10. Despite the current study being underpowered, meaning that the results presented in Table 4. need to be interpreted with caution, these power calculation results provide information that can be used in future research regarding this topic.

Currently, the Dutch Association of Psychiatry does not recommend pre-treatment genotyping in depression. Clinicians could consider doing this in patients who have experienced side effects or inefficacy with psychotropic drugs, including ADs [50]. However, recent RCT data indicated that patients receiving pharmacogenomics (PGx)-guided therapy were more likely to experience symptom remission, less side effects, and/or attain therapeutic plasma concentrations relatively faster, compared with patients who did not receive PGx-guided therapy [51–53]. For example, Swen et al. (2023) indicated that, based on a pre-emptive genotyping strategy using a 12-gene pharmacogenetic panel, PGx-guided therapy (*n* = 725) significantly reduced the incidence of clinically relevant side effects compared with patients without PGx-guided therapy (*n*= 833) [51]. However, these results are not based on AD therapy specifically, but on many other drugs as well. Another RCT by Vos et al. (2023) indicated that PGx-guided dosing of TCAs led to relatively faster attainment of therapeutic plasma concentrations as well as to less side effects, when compared with treatment as usual. However, it is unclear whether similar results can be achieved for other AD groups with less clear concentration-effect/side effect relationships. Furthermore, no significant reduction was seen in depressive symptoms [52]. With regard to the latter, a meta-analysis of five RCTs examining the effect of PGx-guided therapy on remission of depressive symptoms, showed that PGx-guided therapy with ADs had a positive effect on the likelihood of achieving remission in depressive symptoms. This effect may, however, be reserved for individuals with moderate to severe depression and where previous treatment with AD was ineffective or intolerable [53]. This underlines the current recommendation of the Dutch Association of Psychiatry to consider genotyping patients who have experienced side effects or inefficacy of psychotropic drugs [50].

The fact that no significant association was found in the current study between CYP metabolizer status, switching/discontinuing AD treatment and side effects in the hypothesised direction, aligns with the recommendation that genotyping is not indicated in psychiatry as a standard procedure prior treatment. However, considering the design of this study and its limitations we would recommend future studies to include several aspects to assess the best way to implement genotyping in psychiatric patients. First, similar to Swen et al. (2023) [51], to conduct an interventional study, in which pre-emptive screening is compared with treatment as usual, while focusing on ADs specifically. Second, to include large international patient populations to address the relatively low prevalence of, for example, PM and UM metabolizer status in patients of North-European ancestry. Third, to include dose regimen data based on pharmacy records to control for recall-bias and concomitant use of drugs that can result in phenoconversion. And fourth, to include plasma level determination for those drugs with a relatively clear concentration effect/side effect relationship.

In conclusion, this study showed that *CYP2C19* and *CYP2D6* metabolizer status was not significantly associated with switching or discontinuing AD over a 9-year period. In addition, the number of perceived total, serotonergic, cholinergic, and histaminergic side effects did not significantly differ between metabolizer statuses. Additional studies with a larger number of PM and UM patients are needed to more thoroughly investigate these associations and to further determine the potential added value of pharmacogenetic testing to guide pharmacotherapy.

### Supplementary Information

**Supplementary Material 1.**

**Supplementary Material 2.**

**Supplementary Material 3.**

**Supplementary Material 4.**

**Supplementary Material 5.**

**Supplementary Material 6.**

**Supplementary Material 7.**

## Data Availability

According to European data protection law (GDPR), use of data containing potentially identifying or sensitive patient information is restricted; our data involving clinical participants are not freely available in a public repository. However, under certain conditions, data are available upon request via the NESDA Data Access Committee (nesda@ggzingeest.nl) and/or from danielle.cath@ggzdrenthe.nl. See also the website: www.nesda.nl.

## Abbreviations

AD: Antidepressant drug
CPIC: Clinical Pharmacogenetics Implementation Consortium
CYP: Cytochrome P450
DPWG: Dutch Pharmacogenetics Working Group
IM: Intermediate metabolizer
NESDA: Netherlands Study of Depression and Anxiety
NM: Normal metabolizer
PM: Poor metabolizer
SNP: Single Nucleotide Polymorphism
UM: Ultrarapid metabolizer
