# Supplementary material for: Association of CYP2D6 and CYP2C19 metabolizer status with switching and discontinuing antidepressant drugs: an exploratory study
Source: BMC Psychiatry. 2024 May 27;24:394. doi: 10.1186/s12888-024-05764-6 (PMC11129450; doi:10.1186/s12888-024-05764-6)
Supplement: Supplementary file 4 — Supplementary Material 4. [file 12888_2024_5764_MOESM4_ESM.pdf]

	Observation period (in years) →										
	Prior to baseline (T=-3) – (T=0)	Baseline (T=0)	(T=0) – (T=1)	Follow-up (T=1)	(T=1) – (T=2)	Follow-up (T=2)	(T=2) – (T=4)	Follow-up (T=4)	(T=4) – (T=6)	Follow-up (T=6)	Follow-up (T=9)
**Time period**	**1**	**2**	**3**	**4**	**5**	**6**	**7**	**8**	**9**	**10**	**11**
**Patient definition**: Switched	**Drug A**	**X**	**X**	**Drug B**	**X**	**X**	**X**	**X**	**X**	**X**	**X**
Switched	**X**	**X**	**X**	**X**	**X**	**X**	**Drug A**	**Drugs A + B**	**X**	**X**	**X**
Discontinued	**X**	**X**	**X**	**Drug A**	**X**	**X**	**X**	**X**	**X**	**X**	**X**
Discontinued	**Drug A**	**X**	**X**	**Drug A**	**X**	**X**	**X**	**X**	**X**	**X**	**X**
Maintained	**X**	**X**	**X**	**X**	**X**	**X**	**Drug A**	**Drug A**	**X**	**X**	**X**
Maintained	**X**	**X**	**Drug A**	**X**	**X**	**Drug A**	**X**	**X**	**X**	**X**	**X**

Supplementary Figure 1, Additional File 4: *A schematic illustration of criteria for the definition of switched, discontinued, or maintained AD treatment during the observation period.*
The red dotted line (T=1) indicates the timeline essential for the interpretation of criteria 1 and 2 of the patient definitions "maintained on AD" and "discontinued", respectively.
The choice to set the timeline at 'T=1' is based on a suggested AD treatment duration of 6-12 months after a first or recurrent depressive episode, as stated by the Dutch clinical practice guideline for depression.
