# Supplementary material for: Association of CYP2D6 and CYP2C19 metabolizer status with switching and discontinuing antidepressant drugs: an exploratory study
Source: BMC Psychiatry. 2024 May 27;24:394. doi: 10.1186/s12888-024-05764-6 (PMC11129450; doi:10.1186/s12888-024-05764-6)
Supplement: Supplementary file 5 — Supplementary Material 5. [file 12888_2024_5764_MOESM5_ESM.pdf]

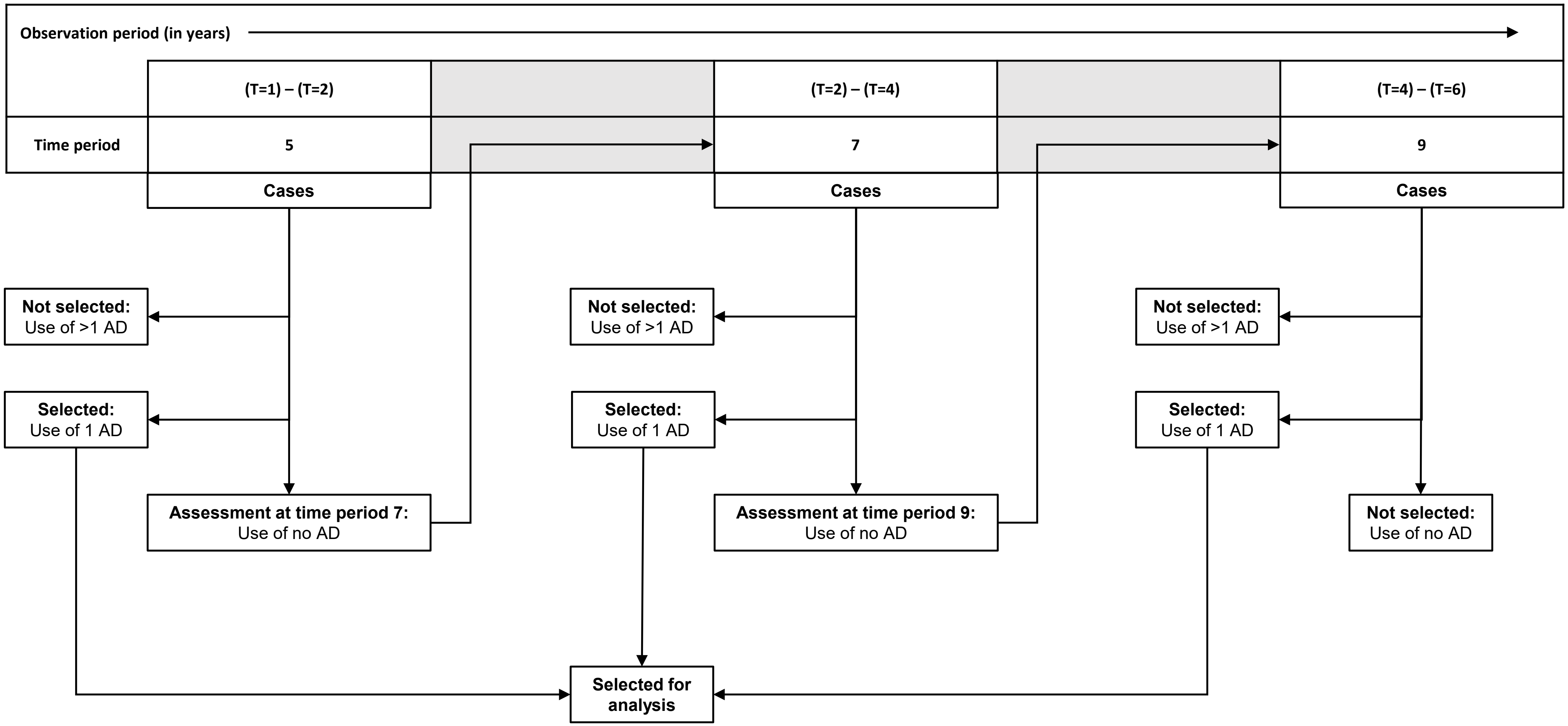

Supplementary Figure 2, Additional File 5: Patient selection process to enable categorization of patients into single AD subgroups.
The figure illustrates the selection process at 3 time periods to enable categorization of patients into single AD subgroups to investigate the prevalence of various reasons to switch or discontinue ADs. The same selection process was conducted for the selection of the study sample, only to take place at all time periods (1 through 10).
