# Supplementary material for: Association of CYP2D6 and CYP2C19 metabolizer status with switching and discontinuing antidepressant drugs: an exploratory study
Source: BMC Psychiatry. 2024 May 27;24:394. doi: 10.1186/s12888-024-05764-6 (PMC11129450; doi:10.1186/s12888-024-05764-6)
Supplement: Supplementary file 7 — Supplementary Material 7. [file 12888_2024_5764_MOESM7_ESM.pdf]

Supplementary Material

The current file presents the post-hoc power calculations for the logistic regression analyses that were conducted to investigate an association between:

- *CYP2C19* metabolizer status and discontinuing AD treatment
- *CYP2C19* metabolizer status and switching AD treatment
- *CYP2D6* metabolizer status and discontinuing AD treatment
- *CYP2D6* metabolizer status and switching AD treatment
- *CYP2C19* UM metabolizer status and discontinuing AD treatment (redefined according to the CPIC)
- *CYP2C19* UM metabolizer status and switching AD treatment (redefined according to the CPIC)

CYP2C19 NM vs. UM – Discontinuing
Power calculation – Odds of discontinuing

The following definitions and formulas are needed to perform a post-hoc power calculation:

Probability of discontinuing AD treatment (Y=1) when being a *CYP2C19* NM patient (X=0):
- $\Pr(Y = 1 | \, X = 1) \, H_0 = \, p_1$

Odds ratio (OR) being calculated according to the following formulas:
- $\exp \, (B_0) = \, p_1 \, / \, 1 - p_1$
- $\exp \, (B_0 + \, B_1) = \, p_2 \, / \, 1 - p_2$
- $OR = (\frac{p_2}{1- \, p_2}) \, / \, (\frac{p_1}{1-p_1})$

Proportion of cases for which X=1 (*CYP2C19* UM) within the total sample size (n=161):
- $X \; parm \; \pi = proportion \; of \; cases \; for \; which \; X = 1 \; (CYP2C19 \; \text{UM})$

CYP2C19 IM & PM Combined * Stopper Crosstabulation

Count

		Stopper		
		Gebruiker	Stopper	Total
CYP2C19 IM & PM Combined	NM	120	24	144
	IM/PM	41	7	48
	UM	13	4	17
Total		174	35	209

Figure 1: *Crosstabulation of CYP2C19 metabolizer status and maintained user (Gebruiker) and discontinuing user (Stopper).*

Post-hoc

- $\Pr(Y = 1|\, X = 1)\ H_0 = \ p_1 = \left(\frac{24}{144}\right) = 0.17$
- $OR = \left(\frac{\frac{4}{17}}{\left(1-\left(\frac{4}{17}\right)\right)}\right)/\left(\frac{\frac{24}{144}}{\left(1-\left(\frac{24}{144}\right)\right)}\right) = 1.54$
- $\Pr(Y = 1|\, X = 1)\ H_1 = calculated\ with\ GPower\ using\ "OR"\ and\ "\Pr(Y = 1|\, X = 1)\ H_0"$
- $X\ parm\ \pi = \frac{17}{161} = 0.11$
- $Total\ sample\ size = 144 + 17 = 161$

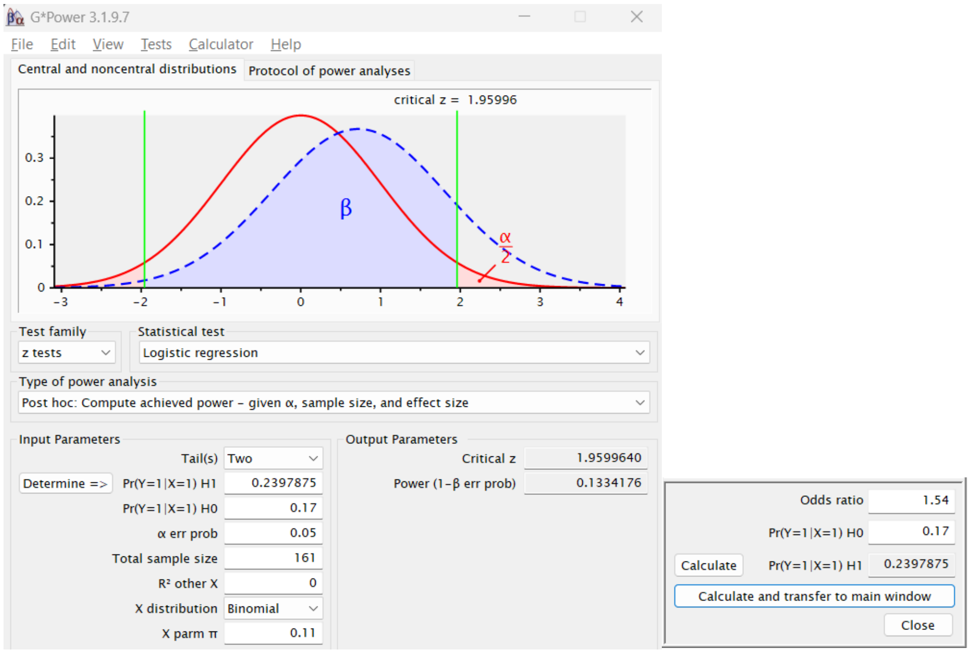

Conclusion:

Based on a total study sample of n=161, the calculated power is 0.13. Compared to a standard power of 0.8, the current study is therefore considered underpowered to investigate an association between *CYP2C19* metabolizer status and discontinuing AD treatment.

***CYP2C19* NM vs. IM/PM – Discontinuing**
Power calculation – Odds of discontinuing

The following definitions and formulas are needed to perform a post-hoc power calculation:

Probability of discontinuing AD treatment (Y=1) when being a *CYP2C19* NM patient (X=0):

- $\Pr(Y = 1 | X = 1)\ H_0 = p_1$

Odds ratio (OR) being calculated according to the following formulas:

- $\exp(B_0) = p_1 / 1 - p_1$
- $\exp(B_0 + B_1) = p_2 / 1 - p_2$
- $OR = (\frac{p_2}{1 - p_2}) / (\frac{p_1}{1 - p_1})$

Proportion of cases for which X=1 (*CYP2C19* IM/PM) within the total sample size (n=192):

- $X\ parm\ \pi = proportion\ of\ cases\ for\ which\ X = 1\ (CYP2C19\ \mathrm{IM/PM})$

CYP2C19 IM & PM Combined * Stopper Crosstabulation

Count

		Stopper		
		Gebruiker	Stopper	Total
CYP2C19 IM & PM Combined	NM	120	24	144
	IM/PM	41	7	48
	UM	13	4	17
Total		174	35	209

Figure 2: *Crosstabulation of CYP2C19 metabolizer status and maintained user (Gebruiker) and discontinuing user (Stopper).*

Post-hoc

- $\Pr(Y = 1 | X = 1)\ H_0 = p_1 = \left(\frac{24}{144}\right) = 0.17$
- $OR = \left(\frac{\frac{7}{48}}{\left(1-\left(\frac{7}{48}\right)\right)}\right) / \left(\frac{\frac{24}{144}}{\left(1-\left(\frac{24}{144}\right)\right)}\right) = 0.85$
- $\Pr(Y = 1 | X = 1)\ H_1 = calculated\ with\ GPower\ using\ "OR"\ and\ "\Pr(Y = 1 | X = 1)\ H_0"$
- $X\ parm\ \pi = \frac{48}{192} = 0.25$
- $Total\ sample\ size = 144 + 48 = 192$

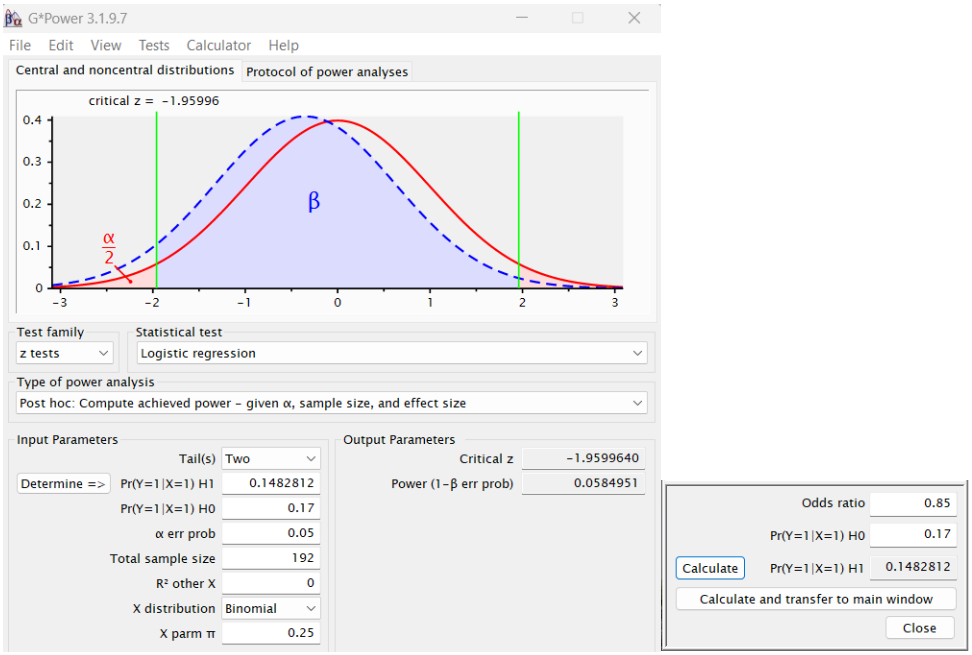

Conclusion:

Based on a total study sample of n=192, the calculated power is 0.06. Compared to a standard power of 0.8, the current study is therefore considered underpowered to investigate an association between *CYP2C19* metabolizer status and discontinuing AD treatment.

CYP2C19 NM vs. UM – Switching
Power calculation – Odds of switching

The following definitions and formulas are needed to perform a post-hoc power calculation:

Probability of switching AD treatment (Y=1) when being a *CYP2C19* NM patient (X=0):
- $\Pr(Y = 1| X = 1)\ H_0 = p_1$

Odds ratio (OR) being calculated according to the following formulas:
- $\exp\ (B_0) = p_1 / 1 - p_1$
- $\exp\ (B_0 + B_1) = p_2 / 1 - p_2$
- $OR = (\frac{p_2}{1- p_2}) / (\frac{p_1}{1-p_1})$

Proportion of cases for which X=1 (*CYP2C19* UM) within the total sample size (n=209):
- $X\ parm\ \pi = proportion\ of\ cases\ for\ which\ X = 1\ (CYP2C19\ \mathrm{UM})$

CYP2C19 IM & PM Combined * Switch_algemeen Crosstabulation

Count

		Switch_algemeen		
		Gebruiker	Switcher	Total
CYP2C19 IM & PM Combined	NM	120	67	187
	IM/PM	41	28	69
	UM	13	9	22
Total		174	104	278

Figure 3: *Crosstabulation of CYP2C19 metabolizer status and maintained user (Gebruiker) and switching user (Switcher).*

Post-hoc

- $\Pr(Y = 1|\,X = 1)\ H_0 = p_1 = \left(\frac{67}{187}\right) = 0.36$
- $OR = \left(\frac{\frac{9}{22}}{\left(1-\left(\frac{9}{22}\right)\right)}\right) / \left(\frac{\frac{67}{187}}{\left(1-\left(\frac{67}{187}\right)\right)}\right) = 1.24$
- $\Pr(Y = 1|\,X = 1)\ H_1 = calculated\ with\ GPower\ using\ "OR"\ and\ "\Pr(Y = 1|\,X = 1)\ H_0"$
- $X\ parm\ \pi = \frac{22}{209} = 0.11$
- $Total\ sample\ size = 187 + 22 = 209$

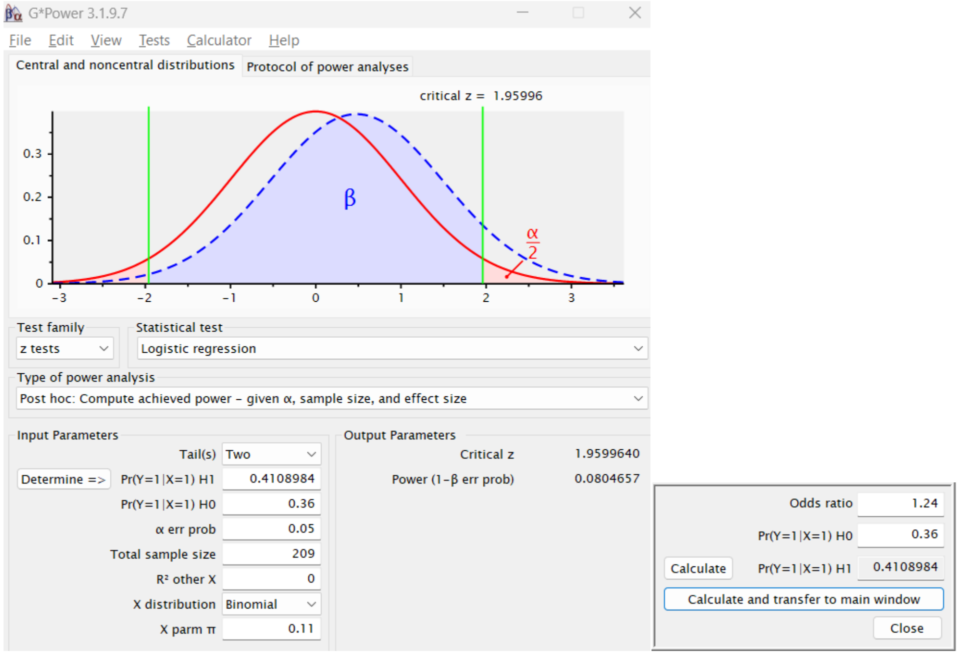

Conclusion:

Based on a total study sample of n=209, the calculated power is 0.08. Compared to a standard power of 0.8, the current study is therefore considered underpowered to investigate an association between *CYP2C19* metabolizer status and switching AD treatment.

CYP2C19 NM vs. IM/PM – Switching
Power calculation – Odds of switching

The following definitions and formulas are needed to perform a post-hoc power calculation:

Probability of switching AD treatment (Y=1) when being a *CYP2C19* NM patient (X=0):

- $\Pr(Y = 1 | X = 1)\ H_0 = p_1$

Odds ratio (OR) being calculated according to the following formulas:

- $\exp\,(B_0) = p_1 \,/\, 1 - p_1$
- $\exp\,(B_0 + B_1) = p_2 \,/\, 1 - p_2$
- $OR = (\frac{p_2}{1 - p_2}) \,/\, (\frac{p_1}{1 - p_1})$

Proportion of cases for which X=1 (*CYP2C19* IM/PM) within the total sample size (n=256):

- $X\ parm\ \pi = proportion\ of\ cases\ for\ which\ X = 1\ (CYP2C19\ \mathrm{IM/PM})$

CYP2C19 IM & PM Combined * Switch_algemeen Crosstabulation

Count

		Switch_algemeen		
		Gebruiker	Switcher	Total
CYP2C19 IM & PM Combined	NM	120	67	187
	IM/PM	41	28	69
	UM	13	9	22
Total		174	104	278

Figure 4: *Crosstabulation of CYP2C19 metabolizer status and maintained user (Gebruiker) and switching user (Switcher).*

Post-hoc

- $\Pr(Y=1|\,X=1)\ H_0 = \ p_1 = \left(\frac{67}{187}\right) = 0.36$
- $OR = \left(\frac{\frac{28}{69}}{\left(1-\left(\frac{28}{69}\right)\right)}\right) / \left(\frac{\frac{67}{187}}{\left(1-\left(\frac{67}{187}\right)\right)}\right) = 1.22$
- $\Pr(Y=1|\,X=1)\ H_1 = calculated\ with\ GPower\ using\ "OR"\ and\ "\Pr(Y=1|\,X=1)\ H_0"$
- $X\ parm\ \pi = \frac{69}{256} = 0.27$
- $Total\ sample\ size = 187 + 69 = 256$

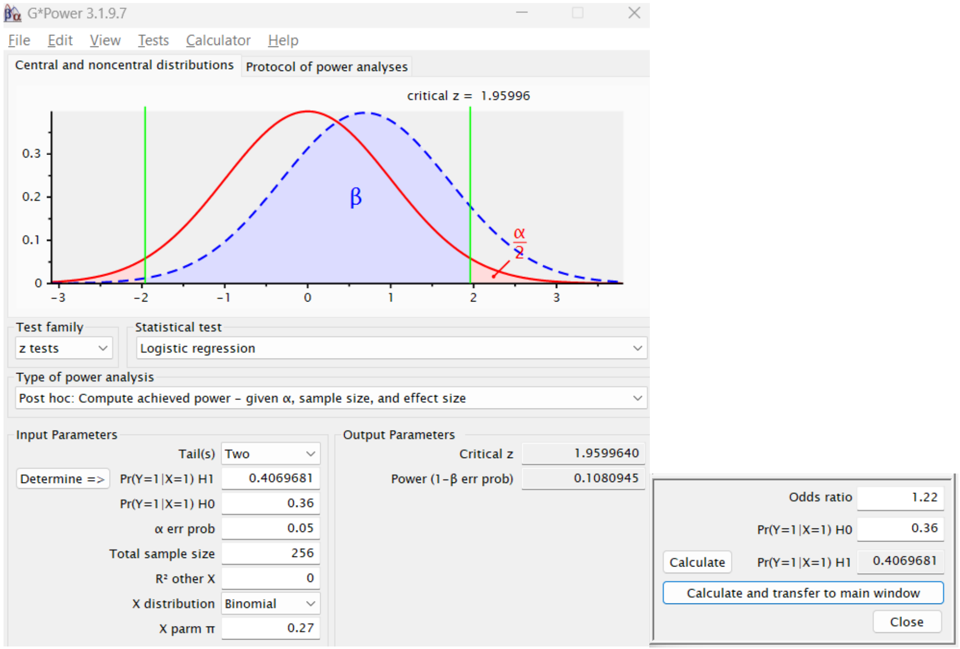

Conclusion:

Based on a total study sample of n=256, the calculated power is 0.11. Compared to a standard power of 0.8, the current study is therefore considered underpowered to investigate an association between *CYP2C19* metabolizer status and switching AD treatment.

***CYP2D6* NM vs. IM/PM – Discontinuing**
Power calculation – Odds of discontinuing

The following definitions and formulas are needed to perform a post-hoc power calculation:

Probability of discontinuing AD treatment (Y=1) when being a *CYP2D6* NM patient (X=0):
- $\Pr(Y = 1 | X = 1)\ H_0 = p_1$

Odds ratio (OR) being calculated according to the following formulas:
- $\exp\,(B_0) = p_1 \,/\, 1 - p_1$
- $\exp\,(B_0 + B_1) = p_2 \,/\, 1 - p_2$
- $OR = (\frac{p_2}{1 - p_2}) \,/\, (\frac{p_1}{1 - p_1})$

Proportion of cases for which X=1 (*CYP2D6* IM/PM) within the total sample size (n=522):
- $X\ parm\ \pi = proportion\ of\ cases\ for\ which\ X = 1\ (CYP2D6\ \mathrm{IM/PM})$

CYP2D6 IM & PM Combined * Stopper Crosstabulation

Count

		Stopper		
		Gebruiker	Stopper	Total
CYP2D6 IM & PM Combined	NM	352	88	440
	IM/PM	60	22	82
Total		412	110	522

Figure 9: *Crosstabulation of CYP2D6 metabolizer status and maintained user (Gebruiker) and discontinuing user (Stopper).*

Post-hoc

- $\Pr(Y = 1|\, X = 1)\; H_0 = \; p_1 = \left(\frac{88}{440}\right) = 0.20$
- $OR = (\frac{\frac{22}{82}}{\left(1 - \left(\frac{22}{82}\right)\right)}) / (\frac{\frac{88}{440}}{\left(1 - \left(\frac{88}{440}\right)\right)}) = 1.47$
- $\Pr(Y = 1|\, X = 1)\; H_1 = calculated\ with\ GPower\ using\ "OR"\ and\ "\Pr(Y = 1|\, X = 1)\; H_0"$
- $X\ parm\ \pi = \frac{82}{522} = 0.16$
- $Total\ sample\ size = 440 + 82 = 522$

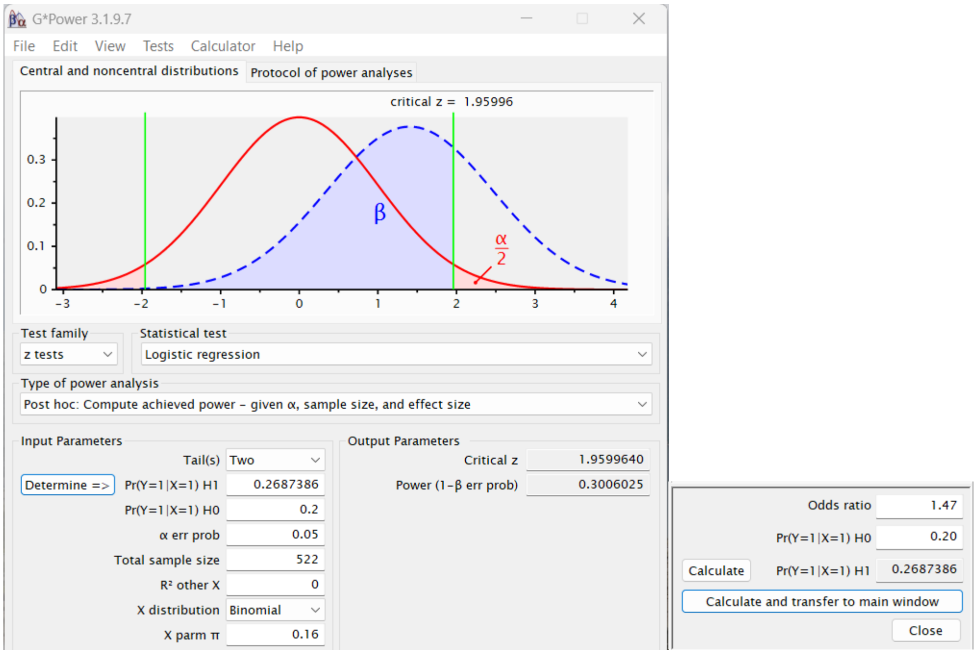

Conclusion:

Based on a total study sample of n=522, the calculated power is 0.27. Compared to a standard power of 0.8, the current study is therefore considered underpowered to investigate an association between *CYP2D6* metabolizer status and discontinuing AD treatment.

CYP2D6 NM vs. IM/PM – Switching
Power calculation – Odds of switching

The following definitions and formulas are needed to perform a post-hoc power calculation:

Probability of switching AD treatment (Y=1) when being a *CYP2D6* NM patient (X=0):

- $\Pr(Y = 1 | X = 1)\ H_0 = p_1$

Odds ratio (OR) being calculated according to the following formulas:

- $\exp\,(B_0) = p_1 / 1 - p_1$
- $\exp\,(B_0 + B_1) = p_2 / 1 - p_2$
- $OR = (\frac{p_2}{1 - p_2}) / (\frac{p_1}{1 - p_1})$

Proportion of cases for which X=1 (*CYP2D6* IM/PM) within the total sample size (n=686):

- $X\ parm\ \pi = proportion\ of\ cases\ for\ which\ X = 1\ (CYP2D6\ \mathrm{IM/PM})$

CYP2D6 IM & PM Combined * Switch_algemeen Crosstabulation

Count

		Switch_algemeen		
		Gebruiker	Switcher	Total
CYP2D6 IM & PM Combined	NM	352	223	575
	IM/PM	60	51	111
Total		412	274	686

Figure 10 *Crosstabulation of CYP2D6 metabolizer status and maintained user (Gebruiker) and switching user (Switcher).*

Post-hoc

- $\Pr(Y = 1|\, X = 1)\ H_0 = p_1 = \left(\frac{223}{575}\right) = 0.39$
- $OR = \left(\frac{\frac{51}{111}}{\left(1-\left(\frac{51}{111}\right)\right)}\right) / \left(\frac{\frac{223}{575}}{\left(1-\left(\frac{223}{575}\right)\right)}\right) = 1.34$
- $\Pr(Y = 1|\, X = 1)\ H_1 = calculated\ with\ GPower\ using\ "OR"\ and\ "\Pr(Y = 1|\, X = 1)\ H_0"$
- $X\ parm\ \pi = \frac{111}{686} = 0.16$
- $Total\ sample\ size = 575 + 111 = 686$

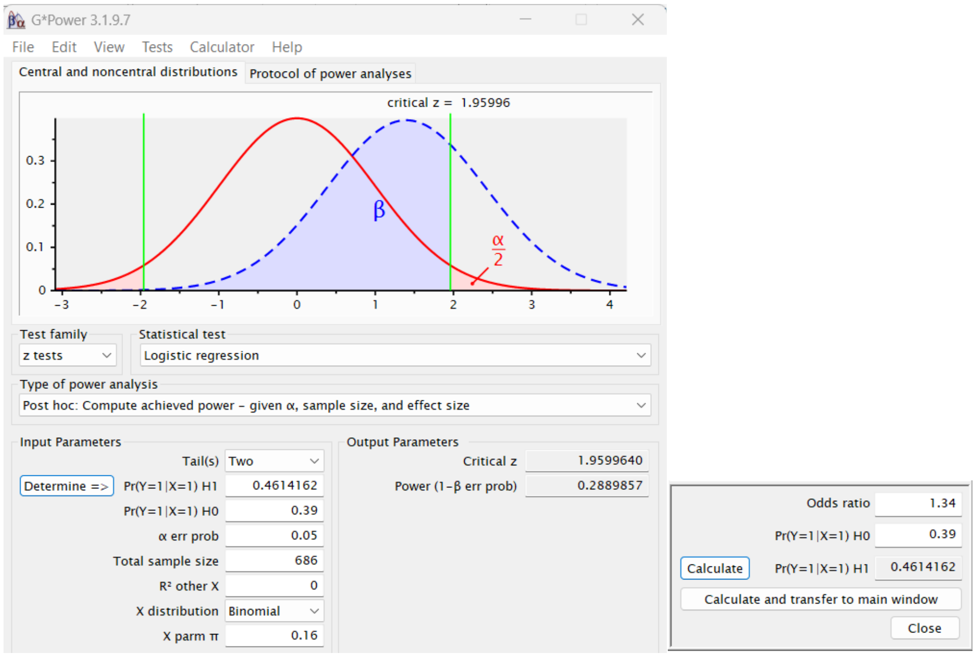

Conclusion:

Based on a total study sample of n=686, the calculated power is 0.29. Compared to a standard power of 0.8, the current study is therefore considered underpowered to investigate an association between *CYP2D6* metabolizer status and switching AD treatment.

<u>*CYP2C19* NM vs. UM – Discontinuing – Redefined UM category (*1/*17 included)</u>

<u>Power calculation – Odds of discontinuing</u>

The following definitions and formulas are needed to perform a post-hoc power calculation:

Probability of discontinuing AD treatment (Y=1) when being a *CYP2C19* NM patient (X=0):

- $\Pr(Y = 1 | \, X = 1) \; H_0 = \; p_1$

Odds ratio (OR) being calculated according to the following formulas:

- $\exp\,(B_0) = \; p_1 \,/\, 1 - p_1$
- $\exp\,(B_0 + \; B_1) = \; p_2 \,/\, 1 - p_2$
- $OR = (\frac{p_2}{1 - \, p_2}) \,/\, (\frac{p_1}{1 - p_1})$

Proportion of cases for which X=1 (*CYP2C19* UM) within the total sample size (n=161):

- $X \; parm \; \pi = proportion \; of \; cases \; for \; which \; X = 1 \; (CYP2C19 \text{ UM})$

CYP2C19 IM & PM Combined * Stopper Crosstabulation

Count

		Stopper		
		Gebruiker	Stopper	Total
CYP2C19 IM & PM Combined	NM	82	16	98
	IM/PM	41	7	48
	UM	51	12	63
Total		174	35	209

Figure 5: *Crosstabulation of CYP2C19 metabolizer status and maintained user (Gebruiker) and discontinuing user (Stopper).*

Post-hoc

- $\Pr(Y=1|\,X=1)\ H_0 = \ p_1 = \left(\frac{16}{98}\right) = 0.16$
- $OR = \left(\frac{\frac{12}{63}}{\left(1-\left(\frac{12}{63}\right)\right)}\right)/\left(\frac{\frac{16}{98}}{\left(1-\left(\frac{16}{98}\right)\right)}\right) = 1.21$
- $\Pr(Y=1|\,X=1)\ H_1 = calculated\ with\ GPower\ using\ \text{"}OR\text{"}\ and\ \text{"}\Pr(Y=1|\,X=1)\ H_0\text{"}$
- $X\ parm\ \pi = \frac{63}{161} = 0.39$
- $Total\ sample\ size = 98 + 63 = 161$

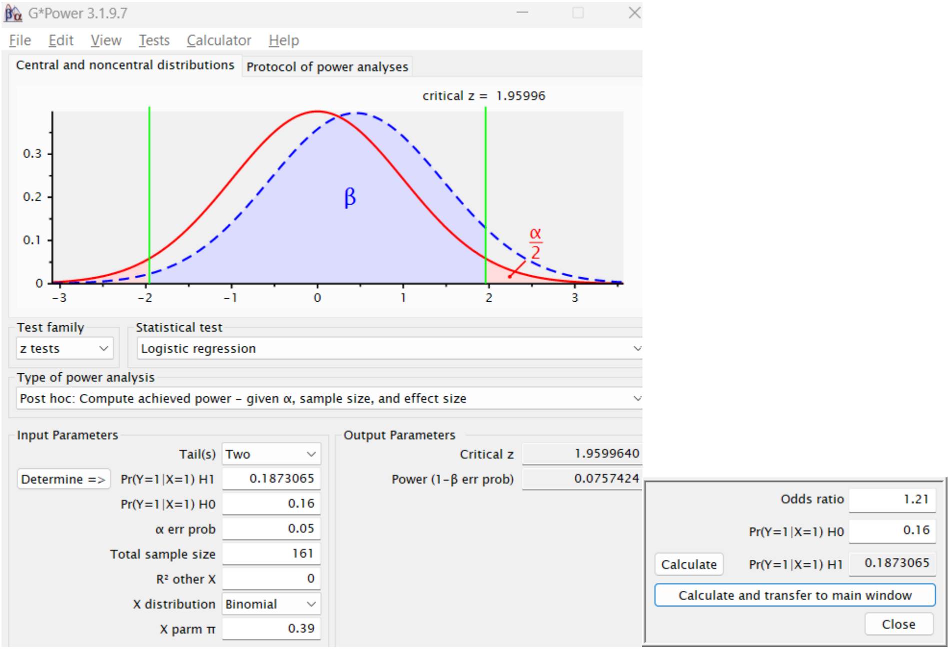

Conclusion:

Based on a total study sample of n=161, the calculated power is 0.08. Compared to a standard power of 0.8, the current study is therefore considered underpowered to investigate an association between *CYP2C19* metabolizer status and discontinuing AD treatment.

CYP2C19 NM vs. IM/PM – Discontinuing – Redefined UM category (*1/*17 included)
Power calculation – Odds of discontinuing

The following definitions and formulas are needed to perform a post-hoc power calculation:

Probability of discontinuing AD treatment (Y=1) when being a *CYP2C19* NM patient (X=0):
- $\Pr(Y = 1 | X = 1)\ H_0 = p_1$

Odds ratio (OR) being calculated according to the following formulas:
- $\exp(B_0) = p_1 / 1 - p_1$
- $\exp(B_0 + B_1) = p_2 / 1 - p_2$
- $OR = (\frac{p_2}{1 - p_2}) / (\frac{p_1}{1 - p_1})$

Proportion of cases for which X=1 (*CYP2C19* IM/PM) within the total sample size (n=146):
- $X\ parm\ \pi = proportion\ of\ cases\ for\ which\ X = 1\ (CYP2C29\ \mathrm{IM/PM})$

CYP2C19 IM & PM Combined * Stopper Crosstabulation

Count

		Stopper		Total
		Gebruiker	Stopper	
CYP2C19 IM & PM Combined	NM	82	16	98
	IM/PM	41	7	48
	UM	51	12	63
Total		174	35	209

Figure 6: *Crosstabulation of CYP2C19 metabolizer status and maintained user (Gebruiker) and discontinuing user (Stopper).*

Post-hoc

- $\Pr(Y = 1 | X = 1)\ H_0 = p_1 = \left(\frac{16}{98}\right) = 0.16$
- $OR = \left(\frac{\frac{7}{48}}{\left(1-\left(\frac{7}{48}\right)\right)}\right) / \left(\frac{\frac{16}{98}}{\left(1-\left(\frac{16}{98}\right)\right)}\right) = 0.88$
- $\Pr(Y = 1 | X = 1)\ H_1 = calculated\ with\ GPower\ using\ "OR"\ and\ "\Pr(Y = 1 | X = 1)\ H_0"$
- $X\ parm\ \pi = \frac{48}{146} = 0.33$
- $Total\ sample\ size = 98 + 48 = 146$

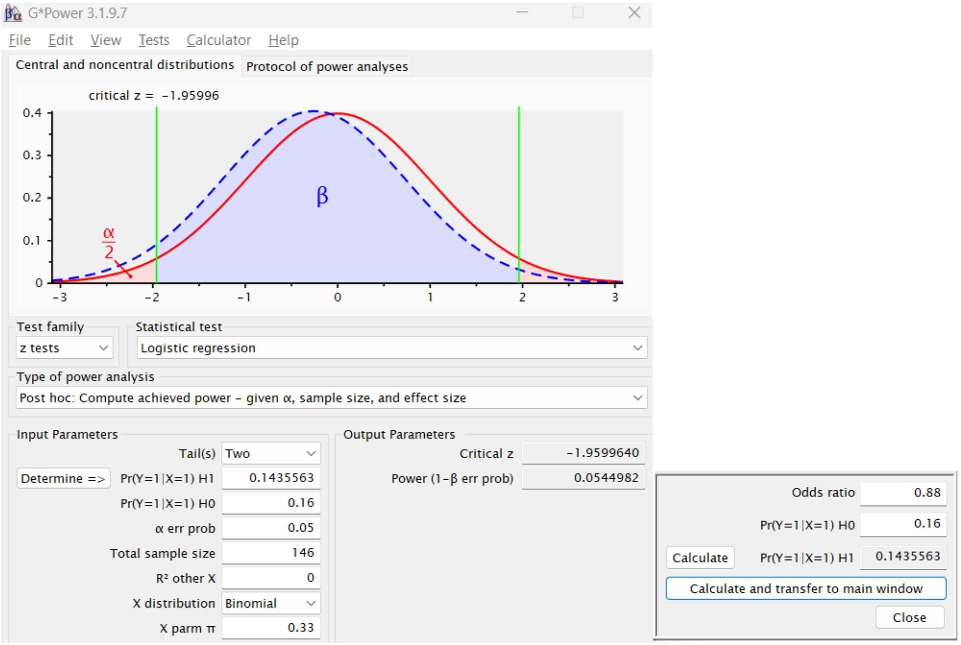

Conclusion:

Based on a total study sample of n=146, the calculated power is 0.05. Compared to a standard power of 0.8, the current study is therefore considered underpowered to investigate an association between *CYP2C19* metabolizer status and discontinuing AD treatment.

CYP2C19 NM vs. UM – Switching – Redefined UM category (*1/*17 included)
Power calculation – Odds of switching

The following definitions and formulas are needed to perform a post-hoc power calculation:

Probability of switching AD treatment (Y=1) when being a *CYP2C19* NM patient (X=0):

- $\Pr(Y = 1 | X = 1)\ H_0 = p_1$

Odds ratio (OR) being calculated according to the following formulas:

- $\exp\,(B_0) = p_1 \,/\, 1 - p_1$
- $\exp\,(B_0 + B_1) = p_2 \,/\, 1 - p_2$
- $OR = (\frac{p_2}{1 - p_2}) \,/\, (\frac{p_1}{1 - p_1})$

Proportion of cases for which X=1 (*CYP2C19* UM) within the total sample size (n=209):

- $X\ parm\ \pi = proportion\ of\ cases\ for\ which\ X = 1\ (CYP2C29\ \text{UM})$

CYP2C19 IM & PM Combined * Switch_algemeen Crosstabulation

Count

		Switch_algemeen		
		Gebruiker	Switcher	Total
CYP2C19 IM & PM Combined	NM	82	46	128
	IM/PM	41	28	69
	UM	51	30	81
Total		174	104	278

Figure 7: *Crosstabulation of CYP2C19 metabolizer status and maintained user (Gebruiker) and switching user (Switcher).*

Post-hoc

- $\Pr(Y = 1 | X = 1)\ H_0 = p_1 = \left(\frac{46}{128}\right) = 0.36$
- $OR = \left(\frac{\frac{30}{81}}{\left(1-\left(\frac{30}{81}\right)\right)}\right) / \left(\frac{\frac{46}{128}}{\left(1-\left(\frac{46}{128}\right)\right)}\right) = 1.05$
- $\Pr(Y = 1 | X = 1)\ H_1 = calculated\ with\ GPower\ using\ "OR"\ and\ "\Pr(Y = 1 | X = 1)\ H_0"$
- $X\ parm\ \pi = \frac{81}{209} = 0.39$
- $Total\ sample\ size = 128 + 81 = 209$

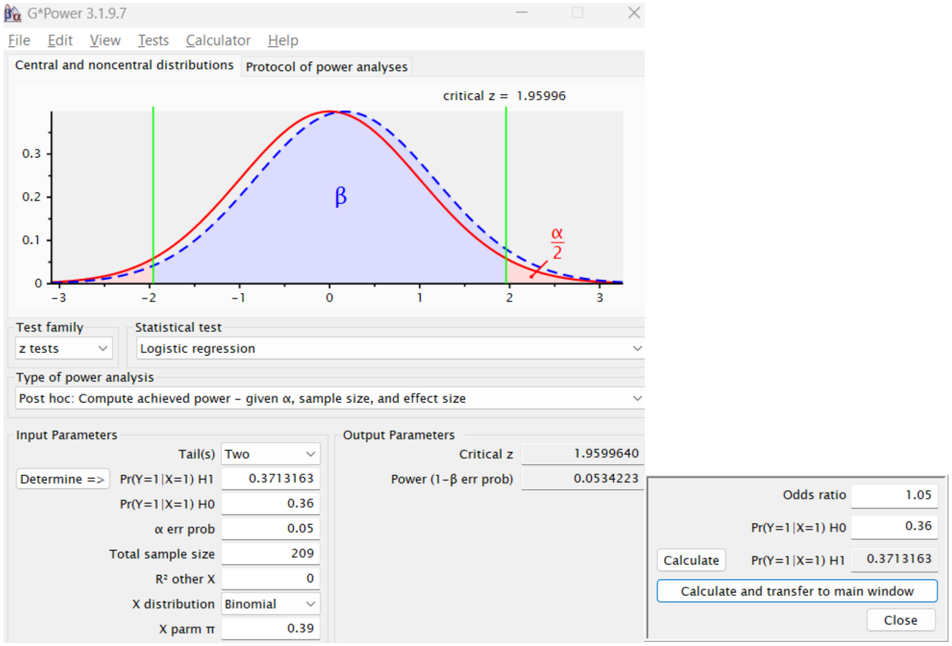

Conclusion:

Based on a total study sample of n=209, the calculated power is 0.05. Compared to a standard power of 0.8, the current study is therefore considered underpowered to investigate an association between *CYP2C19* metabolizer status and switching AD treatment.

***CYP2C19* NM vs. IM/PM – Switching – Redefined UM category (*1/*17 included)**

Power calculation – Odds of switching

The following definitions and formulas are needed to perform a post-hoc power calculation:

Probability of switching AD treatment (Y=1) when being a *CYP2C19* NM patient (X=0):

- $\Pr(Y = 1 | X = 1)\ H_0 = p_1$

Odds ratio (OR) being calculated according to the following formulas:

- $\exp\,(B_0) = p_1 \,/\, 1 - p_1$
- $\exp\,(B_0 + B_1) = p_2 \,/\, 1 - p_2$
- $OR = (\frac{p_2}{1 - p_2}) \,/\, (\frac{p_1}{1 - p_1})$

Proportion of cases for which X=1 (*CYP2C19* IM/PM) within the total sample size (n=197):

- $X\ parm\ \pi = proportion\ of\ cases\ for\ which\ X = 1\ (CYP2C19\ \mathrm{IM/PM})$

CYP2C19 IM & PM Combined * Switch_algemeen Crosstabulation

Count

		Switch_algemeen		
		Gebruiker	Switcher	Total
CYP2C19 IM & PM Combined	NM	82	46	128
	IM/PM	41	28	69
	UM	51	30	81
Total		174	104	278

Figure 8: *Crosstabulation of CYP2C19 metabolizer status and maintained user (Gebruiker) and switching user (Switcher).*

Post-hoc

- $\Pr(Y = 1 | X = 1)\ H_0 = p_1 = \left(\frac{46}{128}\right) = 0.36$
- $OR = \left(\frac{\frac{28}{69}}{\left(1-\left(\frac{28}{69}\right)\right)}\right) / \left(\frac{\frac{46}{128}}{\left(1-\left(\frac{46}{128}\right)\right)}\right) = 1.22$
- $\Pr(Y = 1 | X = 1)\ H_1 = calculated\ with\ GPower\ using\ "OR"\ and\ "\Pr(Y = 1 | X = 1)\ H_0"$
- $X\ parm\ \pi = \frac{69}{197} = 0.35$
- $Total\ sample\ size = 128 + 69 = 197$

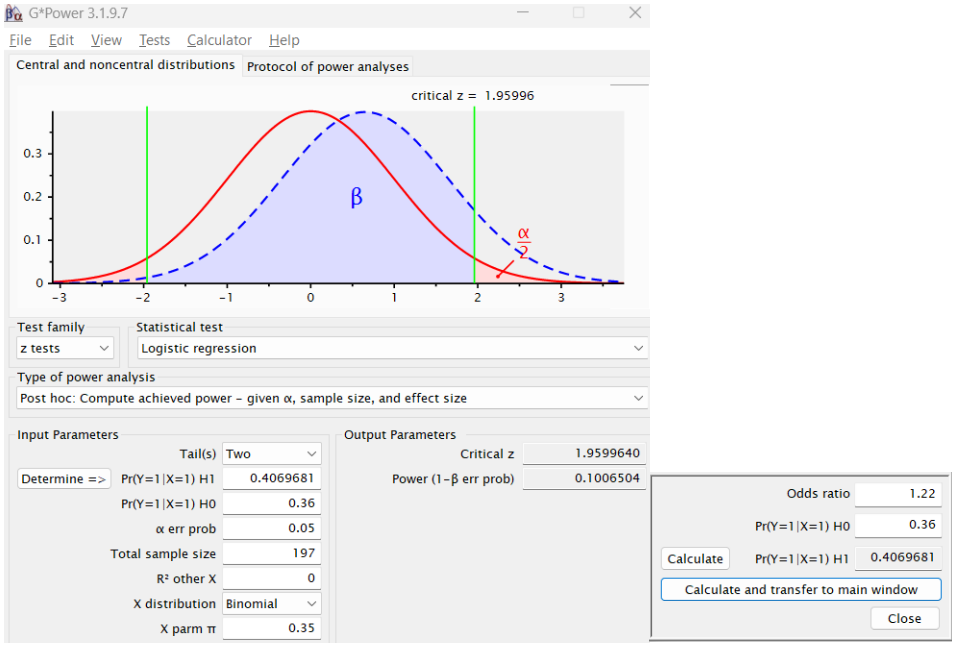

Conclusion:

Based on a total study sample of n=197, the calculated power is 0.10. Compared to a standard power of 0.8, the current study is therefore considered underpowered to investigate an association between *CYP2C19* metabolizer status and switching AD treatment.
